# Virus-Encoded Circular RNAs: Role and Significance in Viral Infections

**DOI:** 10.3390/ijms242216547

**Published:** 2023-11-20

**Authors:** Giuseppe Sberna, Fabrizio Maggi, Alessandra Amendola

**Affiliations:** Laboratory of Virology and Biosafety Laboratories, National Institute for Infectious Diseases “L. Spallanzani” IRCCS, 00149 Rome, Italy; giuseppe.sberna@inmi.it (G.S.);

**Keywords:** circular RNA, circRNA, non-coding RNA, viral infection, biomarkers of infection

## Abstract

Circular RNAs (circRNAs) have been the focus of intense scientific research to understand their biogenesis, mechanisms of action and regulatory functions. CircRNAs are single stranded, covalently closed RNA molecules lacking the 5′-terminal cap and the 3′-terminal polyadenine chain, characteristics that make them very stable and resistant. Synthesised by both cells and viruses, in the past circRNAs were considered to have no precise function. Today, increasing evidence shows that circRNAs are ubiquitous, some of them are tissue- and cell-specific, and critical in multiple regulatory processes (i.e., infections, inflammation, oncogenesis, gene expression). Moreover, circRNAs are emerging as important biomarkers of viral infection and disease progression. In this review, we provided an updated overview of current understanding of virus-encoded and cellular-encoded circRNAs and their involvement in cellular pathways during viral infection.

## 1. Introduction

It has recently been shown that 90% of the eukaryotic genome is transcribed, yet only about 2% is translated. Indeed, most of these transcripts are non-coding forms of RNA (ncRNA). In recent years, these types of RNAs have been the focus of intense scientific research to understand their biogenesis, mechanisms of action and regulatory functions they can and could play at the level of the main biological processes, such as during infections, inflammation and during neoplastic transformation [1,2,3,4,5]. Importantly, ncRNAs have been identified as the main regulators of gene expression. NcRNAs have been divided into three main classes: micro RNAs (miRNAs), long non-coding RNAs (lncRNA) and circular RNAs (circRNAs).

CircRNAs are single stranded, covalently closed RNA molecules lacking the 5’-terminal cap and the 3′-terminal polyadenine chain [6,7]. For a long time, these RNAs were considered to have no precise function and they were believed to be the consequence of defective splicing events. However, recent evidence has led to reconsideration of the value and importance of circRNAs in many species by virtue of their multifunctional nature which sees them involved in several biological and pathological processes. To date, several functions carried out by circRNAs have been identified. For example, they can act as sponges of miRNAs, modulators of RNA transcription and of protein synthesis [8].

The history of circRNAs began in 1976 with Sanger et al. [9], who discovered the structure of plant viroids, which were nothing more than non-coding circRNAs [9]. In the same year, Kolakofsky visualised circRNAs in the Sendai virus with an electron microscope. He defined the viral circRNAs as defective interfering (DI)-RNAs, for their ability to interfere with genome replication of the Sendai virus [10]. The discovery of the hepatitis delta virus (HDV) genome structure as a circular RNA [11] led scientists to become much more interested in the research, discovery and study of this fascinating form of RNAs. Further studies and especially the advent of new sequencing technologies allowed the discovery of a vast number of different archaea [12], plants [9], animals [13] and human circRNAs [14].

Recently, circRNAs encoded by the viruses have also been identified [15,16]. Different viruses, with both DNA and RNA genomes, such as the Epstein–Barr virus (EBV) [15], Kaposi’s sarcoma herpesvirus (KSHV) [16], human papillomavirus 16 (HPV-16) [17], coronaviruses [18] and all the main flaviviruses produce circRNAs and use them to finely regulate processes fundamental for their spread and survival [19].

**Figure 1:**
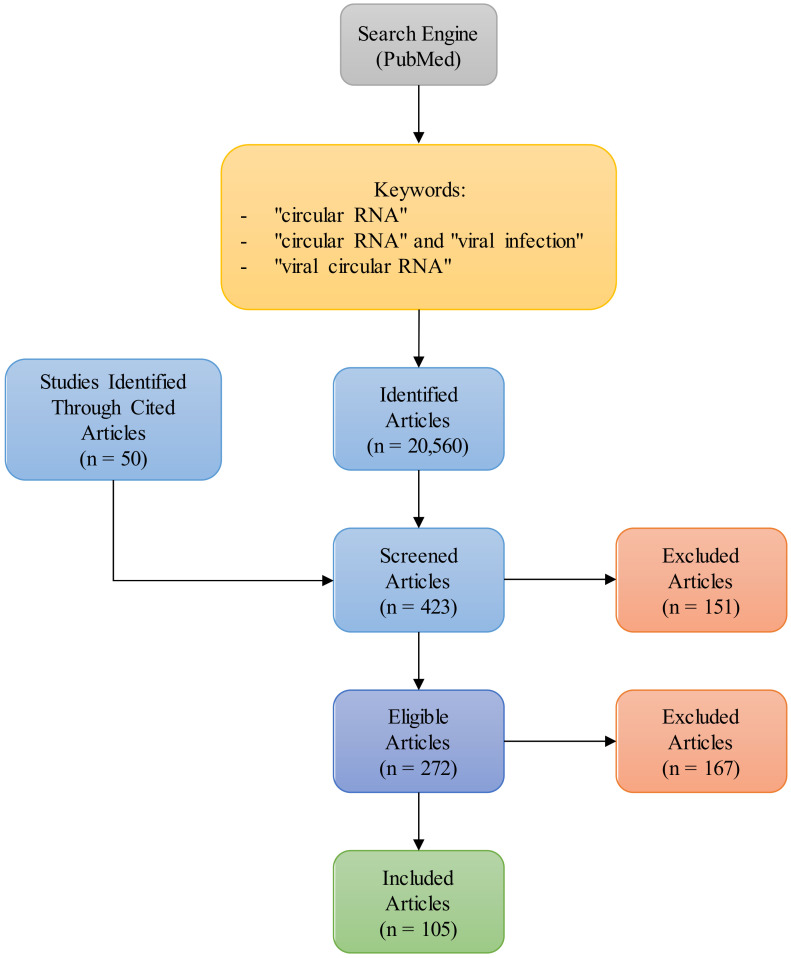
Graphic representation of reference search.

In this review, we provided an overview of the current understanding of virus-encoded circRNAs, with a focus on their function and potential uses in therapies. We also mention cellular-encoded circRNAs that modulate the replicative cycle of viruses during infections. A graphic representation of how the literature search was carried out is shown in Figure 1.

## 2. Biogenesis of circRNAs

The formation and circularisation of circRNAs is a complicated process that is regulated by the spliceosome and by several cis- and trans-acting regulatory elements [20,21].

In eukaryotic cells, spliceosomes catalyse the splicing of precursor mRNAs (pre-mRNAs). During this process, if the upstream 5′ donor splicing site joins the downstream 3′ acceptor splicing site, a linear mRNA is obtained. However, spliceosome can also generate circRNAs during the processing of the pre-mRNA (pre-messenger RNA), the pre-tRNA (pre-translation RNA) and the pre-rRNA (pre-ribosomal RNA) molecules [22]. CircRNAs structure can originate from different components of the genome and based on this, Wang et al. [23] divided circRNAs into three categories:

- Exonic circRNAs (ecircRNAs), the largest group composed only of exons;
- Intronic circRNAs (ciRNAs), composed only of introns;
- Exon–intron circRNAs (elciRNAs), contain both exons and introns.

These categories of circRNAs have been shown to have precise distribution within the cell; ciRNAs and elciRNAs are usually located in the nucleus [24], whereas ecircRNAs are in the cytoplasm [25].

There is another category of non-exonic circRNAs termed “intergenic” [26]. These are formed by two intronic circRNAs fragments flanked by GT-AG splicing signals, which function as donor and acceptor sites of circularised splicing [26].

In addition, another kind of circRNA has been described: the tricRNAs [27]. These circRNAs originate through the removal of introns from pre-tRNAs [28].

Among the processes leading to the formation of circRNAs from pre-mRNAs the best known is certainly the “directed back-splicing” [29,30,31]. Both the two 3′ upstream and 5′ downstream ends link together giving rise to a new circRNA molecule [32,33]. Alternative RNA back-splicing mechanism can also lead to the generation of multiple circRNAs when two or more back-splicing sites at the 5′ or 3′ downstream bind to the same back-splicing site at the 3′ or 5′ upstream in a reverse orientation [29,34].

Another mechanism leading to the production of circRNAs is the “lariat formation” [29,34]. It can take place in two different ways: by “intron-pairing-driven” and by “exon skipping” circularisation [35]. In “intron-pairing-driven” circularisation described for the first time by Jeck et al. [36], the small nuclear RNA U1 cuts the 5′ end of the pre-mRNA and allows the binding of the 5′ and 3′ bases between guanidine and adenosine [31,37,38]. During exon skipping, a “hetero lariat”, namely a structure containing both introns and exons, is produced [31,39]; when introns are removed, the composition of circRNAs contains only exonic sequences [30].

## 3. Identification and Quantification of circRNAs

In the past, identification of circRNAs in RNA sequencing experiments was almost impossible because reads containing back-splicing junctions were not recognised by genome alignment algorithms and could not be mapped [40]. In recent years, the development of high-throughput sequencing and bioinformatics analysis methods has made the detection of highly expressed circRNAs a little bit easier. Today, more than 10,000 circRNAs are known in the animal world and the number of circRNAs encoded by viruses is constantly growing [41].

For both the identification and the de novo detection of viral circRNAs, it is now possible to use high-throughput genome-wide RNA sequencing analysis (RNA-Seq) [40,42]. RNA-seq consists in a process of creating short sequencing reads from RNA molecules. The procedure requires converting the RNA into cDNA, then (optionally) amplifying the cDNA by PCR and finally dissecting the cDNA into short fragments. The fragments are used as input for next-generation sequencing. The resulting sequence reads are then aligned to a reference genome. The enormous amount of raw data to be analysed requires time and resources, such as highly specialised personnel capable of dealing with the inherent limitations of sequencing technologies and appropriate state-of-the-art RNA-seq tools [43].

For the circRNAs identification, different algorithms such as CIRCexplorer2 [44] and CIRI2 [45] are available and are able to specifically detect head-to-tail alignments. To date, although many RNA-seq tools can make easy to recognise and quantify circRNAs, the discrepancy in the analytical approaches not yet standardised remains the major issue for the identification of these special RNAs. The discrepancy can be the consequence of sample selection mode, the technique used for sequencing, the technique of detection and computational algorithms and filters used during analyses of databases. For example, considering the RT-qPCR method, a high variability of results is often obtained. This method involves several steps consisting of RNA extraction followed by treatment with RNAse R to remove linear RNA, cDNA synthesis, purification and quantification of the RNA; however, it is now known that hundreds of linear transcripts fail to be digested by the RNase R because they lack at least seven nucleotides at the 3′ single stranded overhangs necessary for the binding by the RNase R [46]. Another cause of discrepancy concerns the computational algorithms used to identify reads; they may have significant differences in sensitivity and precision, so the identified sequence should at least be confirmed by another algorithm and the presence of the RNA should be validated using a different technique such as RT-PCR or northern blotting [46].

CircRNAs can be detected also by fluorescence in situ hybridisation, aptamers, or BaseScope technology through specific probes [40].

Furthermore, public transcriptome databases are available and can serve in recognising profiles of conserved and abundant circRNAs across species and tissues, with their expression pattern, degree of conservation, and functional annotation [47,48].

## 4. miRNAs: “Partners-in-Crime” of circRNAs

In eukaryotic cells, gene expression is finely regulated by miRNAs, small ncRNA molecules of about 18–22 nucleotides long [49]. The functions performed by miRNAs, in turn, are modulated by circRNAs and also by other RNA species with similar capabilities, such as lncRNAs [50].

The “first step” in the formation of miRNAs takes place in the nucleus of a cell: the pri-miRNA (miRNA precursor) thanks to the enzyme Drosha (RNAseIII). This cleaves the primary transcripts, giving rise to the the pre-miRNA. In the “second step”, the pre-miRNA is exported into the cytoplasm where Dicer (another RNAseIII) produces a short double stranded RNA. One strand binds argonaute proteins (mainly AGO2) to form the miRNA-induced silencing complex called miRISC [51]. The miRNA–mRNA interaction, more specifically miRISC–mRNA, results in mRNA degradation or translation inhibition, leading to reduced gene expression and modulation of related biological function [52].

During viral infections, the cellular miRNAs perform several functions; for example, controlling viral production and viral RNA turnover, blocking virus replication, suppressing viral factors, interacting with viral genome and inhibiting of host antiviral factors [53].

In addition to cellular miRNAs, viral-derived miRNAs (v-miRNAs) have been identified. The v-miRNAs play a role in regulating viral life cycles and host mRNAs functions that support virus replication [53].

Recent research has revealed the intriguing interplay between miRNAs and circRNAs, where the latter demonstrate a remarkable ability to sponge miRNAs, thus, adding complexity to their regulatory functions. An example is the human ciRS-7 which possesses more than 60 binding sites for miR-7 [38,54]. Given its numerous binding sites for miR-7, it has long been assumed that ciRS-7 sequesters miR-7 and prevents it from regulating the expression of target mRNAs [38,54]. However, it was seen that deletion of this circRNA did not lead to a decrease in the levels of most miR-7 target genes [55]. On the contrary, an increase in the expression of some miR-7 target genes was observed. Therefore, the main function of ciRS-7 may be to control the temporal and spatial localisation of the miRNA and/or to protect it from degradation [55,56].

## 5. Functions of circRNAs

Most studies have focused on circRNAs encoded by the cells at various stages of their life, during which they play innumerable and fundamental roles [57], such as miRNA sponges [58], regulation of cellular proliferation [59], protein-binding [60], splicing regulation [61] and roles in the immune system [62]. Due to their involvement in all these crucial functions, it is assumed that cellular circRNAs could have strong correlation with various diseases. These assumptions have been confirmed for several diseases, in which the presence of circRNAs has been linked to the onset of particular pathologies (for a more careful and detailed discussion of cellular-encoded circRNAs that seem to be involved in diseases, please refer to other readings, such as the review by Lei et al. [63]).

### 5.1. Functions of Cellular-Encoded circRNAs Involved in Viral Infections

Studies on circRNAs have led to the identification of numerous cellular-encoded circRNAs involved in viral infections and/or in virus-related cancer diseases. An updated list of cellular-encoded circRNAs is given in Table 1.

In many viral infections, circRNAs of cellular origin appear to be involved via upregulation or downregulation of their levels in infected cells and/or in biological fluids [66,84].

For example, Shi et al. [64] observed significant downregulation of the 348 circRNAs and significant upregulation of the 188 circRNAs in cells infected with herpes simplex virus 1 (HSV-1). In hepatitis B virus (HBV)-associated hepatocellular carcinoma, the hsa_circ_0067934 acts as a sponge for the miR-1324, thus, altering the FZD5/Wnt/β-catenin signalling pathway, which participates in the proliferation, migration, and invasion of the HBV-associated hepatocellular carcinoma [69]. Another circRNA, circRNA_100338, which has been found to be overexpressed still in HBV-associated hepatocellular carcinoma, seems to act as a sponge for miR-41-3p for which it has been suggested as reliable biomarker for the diagnosis of this carcinoma [69]. In cervical carcinoma caused by HPV-16, the hsa_circ_0018289 is over-expressed [75]; by removing these circRNAs it is possible to suppress migration, proliferation and invasion of cervical cancer; thus supporting the role of the hsa_circ_0018289 in the tumorigenesis of cervical cancer [75].

Some cellular-encoded circRNAs have been found to be related to human immunodeficiency virus 1 (HIV-1) infection [81,82]. Specifically, hsa_ANKRD17_0008, hsa_ATXN1_0001, hsa_FAM13B_0019, hsa_FBXW7_0005, hsa_HIPK3_0001, hsa_PHC3_0020, and hsa_ZNF609_0001 are commonly detected in viremic patients, but they are absent in patients after taking antiretroviral therapy (ART) [82]. Also, in the same analysis, some patients prior to ART showed circRNAs that, when involved in the circRNA-miRNA-mRNA networks, could potentially promote viral replication [82]. In effect, this was possible because miRNAs, which have the ability to decrease HIV-1 replication, were sequestered by cellular-encoded circRNAs [82].

Bhardwaj et al. [83] emphasise more the role of cellular-encoded circRNAs during HIV-1 infection. They show that hsa_circ_0001445 (referred as ciTRAN) plays a role in modulating HIV-1 transcription through interaction with viral accessory protein (Vpr). In particular, HIV-1 Vpr hijacks ciTRAN to exclude serine/arginine-rich splicing factor 1 (SRSF1) from the viral transcriptional complex, thereby allowing efficient viral transcription [83].

In human umbilical vein endothelial cells infected with Hantaan virus, following infection, the expression of hsa_circ_0000479 increased, suppressing viral replication by sponging miR-149-5p [58].

In a study by He et al. [84], two cellular circRNAs were identified as possible biomarkers of infection in patients with dengue virus infection. In the peripheral blood mononuclear cells of these patients, hsa_circ_0015962 and hsa_circ_0006459 were significantly upregulated and downregulated, respectively [84]. These differences in their level were observed between the pre-treatment and post-treatment periods. Specifically, the levels of hsa_circ_0015962 and hsa-miR-113b were found to be higher in the post-treatment group than in the pre-treatment group; the opposite was true for hsa_circ_0006459 and its complementary hsa-miR-4683, [84]. Indeed, when hsa_circ_0015962 binds hsa-miR-4683 and hsa_circ_0006459 binds hsa-miR-133b their activity is hindered. He et al. [84] speculated that the interactions between circRNAs and miRNAs might provide new input for research into the pathological mechanisms and in treatment response to dengue virus.

Certain circRNAs seem to influence also the autophagy in infected cells. Autophagy is a catabolic process related to protein and organelle degradation and is essential for maintaining cellular homeostasis [87]. In addition, autophagy is a process actively involved in viral infectiousness as it may both stimulate or inhibit the replication of viruses by playing a role in modulating cell survival [88]. Some viruses have developed the ability to alter levels of specific circRNAs to regulate autophagy. For example, the knockdown of circ-GATAD2A within A549 cells infected with influenza A virus (i.e., H1N1) suppresses viral replication, promoting autophagy instead. The exact opposite occurs by upregulating circ-GATAD2A in H1N1-infected A549 cells, which promotes viral replication by blocking autophagy. Hence, circ-GATAD2A has the capacity to block autophagy and to promote viral replication [79]. The regulation of autophagy by circRNAs, confirmed in other in vitro models of viral infection, deserves to be included among new therapeutic purposes in development.

In the human lung adenocarcinoma cells infected with Middle East respiratory syndrome coronavirus (MERS-CoV), the expression of hsa_circ_0067985 and hsa_circ_0006275 is upregulated [86]. Of significance is the fact that the knockdown of these circRNAs reduced the cellular viral load and decreased the expression of target genes through the modulation of MAPK and the ubiquitination pathways [86].

### 5.2. Functions of Virus-Encoded circRNAs

As mentioned above, viruses can also produce circRNAs (Table 2) and, interestingly, these molecules perform functions similar to those produced by cells. This is even more true when considering the similar structure, generation, and conservation among species of circRNAs derived from DNA or RNA viruses compared with those of host-encoded circRNAs.

Many studies have been carried out to discover, identify and understand whether and how these circRNAs encoded by viruses could be exploited as biomarkers, perhaps even prognostic, of infection and probably be as useful as in therapy.

CircE7, identified in HPV-16 positive cervical cancer cell lines, is encoded by the same virus, and formed from E6 and E7 pre-mRNA by back-splicing. This circRNA was shown to be translated into the E7 oncoprotein [89].

The HBV infection is a key risk factor for hepatocellular carcinoma. HBV contributes to the development of hepatocellular carcinoma through its DNA integration into the host genome by inducing clonal expansion of infected cells and by favouring the genomic instability; moreover, the sustained expression of viral proteins (such as HBV X protein) can influence cell death, proliferation and signalling pathways in favour of the viral infection [98]. Among the viral factors, Zhu et al. [90] identified HBV_circ_1, which derives from HBV pre-genomic RNA, an element that seems to play a role in viral latency and replication. In fact, HBV_circ_1 was found at higher levels in hepatocellular carcinoma tissues than in paracancerous tissues [90]. In the same study, it was seen that HBV_circ_1 induces cell proliferation and migration, blocks apoptosis in vitro, promotes Ki67 and cyclin D1 expression in the formed tumour tissue and increases tumour size in vivo [90]. It was also found that in HBV_circ_1-negative patients the survival rate was significantly higher than that observed in HBV_circ_1-positive patients; this important finding seems to confirm that the HBV_circ_1 contributes to HBV-related tumour progression [90].

As shown in Table 2, EBV is another virus that produces circRNAs. Previous studies have shown that EBV contributes to the pathogenesis of EBV-associated diseases through coding and non-coding genes involved in latent and lytic cycles [99,100]. These genes express a repertoire of circRNAs, in which investigation of their prevalence and biological roles has only begun recently. In a study by Ge et al. [91], the EBV-produced circBART2.2 was found at high concentrations in nasopharyngeal carcinoma (where EBV presence plays a significant role). The circBART2.2 is correlated both with the inhibition of T-cell function and the upregulation of the programmed cell death-ligand 1, which belong to a signalling pathway mediating tumour immunosuppression, thus, favouring the carcinoma proliferation. In particular, circBART2.2 promotes the transcription of PD-L1 in carcinoma cells allowing them to hinder their recognition by the immune system through the PD-1/PD-L1 checkpoint. The transcription of PD-L1 is induced when circBART2.2 binds RIG-I and activates the transcription factors IRF3 and NF-κB [91].

In another study on nasopharyngeal carcinoma [92], a new circRNA has been identified, the circLMP-2_e5. It is co-expressed with the linear RNA LMP-2 when EBV is reactivated in the lytic phase. Because the expression pattern of circLMP-2_e5 appears to resemble that of its comparable linear LMP-2 gene, it could be the result of exon-skipping and circularisation of its linear equivalent. This process may occur without the need for Alu repeats or non-repetitive inverted complementary sequences in the flanking introns. The circLMP-2_e5 has been localised not only into the nucleus of EBV-infected cell lines (where it may modulate transcriptional processes in the host cells), but also into the cytoplasm, where it may act as a miRNA or an RNA binding protein (RBP) sponge or a template for translation. Interestingly, this circRNA does not appear to be involved in the recognition of the host’s innate immune response, cell proliferation, lytic reactivation of EBV or its parental linear transcripts [92]. Additional analyses should investigate if circLMP-2_e5 operates as a miRNA or RBP sponge, regulates other transcription processes, or is a by-product of the pre-mRNA splicing [92].

Furthermore, circRPMS1, also encoded by EBV, reaches higher levels in metastatic nasopharyngeal carcinoma [93]. Compared to circLMP-2_e5 which did not appear to be involved in regulatory processes [92], the knockdown of circRPMS1 inhibited cell proliferation, induced apoptosis, and repressed cell invasion of carcinoma cells [93]. Consequently, this confirms that circRPMS1 promotes cellular oncogenesis by sponging multiple miRNAs [93].

Gong et al. [94] demonstrated that analogous to circRPMS1, the EBV-encoded circLMP2A is capable to promote the stemness phenotype in EBV-associated gastric cancer cell lines by overcoming the miR-3908/TRIM59/p53 axis through the sponging of miR-3908 [94]. In line with these findings, in clinical samples of EBV-associated gastric cancer, the levels of circLMP2A and miR-3908 were negatively correlated [94]. It should also be noted that both metastases produced by EBV-associated gastric cancer and poor prognosis have been positively correlated with the expression of circLMP2A [94].

Recently, scientific interest has focused on human coronaviruses due to the recent pandemic [101]. CircRNAs have been included in scientific research and attempts have been made to identify circRNAs that are produced by these pathogens and may be involved in their life processes. For instance, Yang et al. [102] were able to identify 224, 351 and 2764 circRNAs produced by the severe acute respiratory syndrome coronavirus (SARS-CoV), SARS-CoV-2 and MERS-CoV, respectively [102]. In another study by Cai et al. [18], they were able to identify 720, 3437 and 28754 circRNAs also produced by SARS-CoV, SARS-CoV-2 and MERS-CoV, respectively [18]. To date, the pattern of expression and their respective miRNA membership axes are not yet known and are being studied intensively.

## 6. Therapeutic Approaches Involving circRNAs in Viral Infection

In virus-infected cells, circRNAs could affect host protein-coding mRNAs by exploiting the host’s miRNA network, given their function as sponges for ncRNAs. As circRNAs can target viral miRNAs and inactivate them, they hold a high potential as antiviral agents, so they are currently under the spotlight and extensively studied in developing treatment strategies for the management of viral infections.

For example, as previously mentioned above, the knockdown of two circRNAs (hsa_circ_0067985 and hsa_circ_0006275) in human lung epithelial cells infected with MERS-CoV induces a significant decrease in viral load [86]. During Hantaan virus infection, a substantial reduction in viral load was found following the level increase of hsa_circ_0000479, a circRNA sponging the miR-149-5p [58].

Another example demonstrating the powerful role of circRNA as an antiviral agent is observed in HBV-associated hepatocellular carcinoma tissues and cells. Here, elevated levels of tumour necrosis factor-induced protein 3 and hsa_circ_0006942 correlate with miR-138-5p downregulation. This evidence suggests a possible way to exploit circRNAs, which upregulate the expression of an antiviral component by sponging specific miRNA [67]. Likewise, downregulation of circRNA_10156, a circRNA highly expressed in HBV-related liver cancer [73], involved in the circRNA_10156/miR-149-3p/AKT1 signalling pathway [73], leads to increased expression of miR-149-3p, thereby depleting the expression of Akt1 and preventing the proliferation of liver cancer cells [73].

Again, during the HSV-1 infection, cellular responses involve many immunity-related genes including the circRNA-miRNA-regulatory axis, mainly those enriched in the NOD-like receptor/JAK-STAT signalling pathway, which becomes dysregulated because of altered circRNA expression [64].

Other examples of circRNAs that may be used for therapeutic purposes are those mentioned above originating from EBV (circRPMS1 and ebv-circLMP2A) [93,94] and H1N1 (circ-GATAD2A) [79].

Wang et al. [103] demonstrated that circ_chr19, sponging miR-30b produced by the Ebola virus, can help the immune system to identify and inhibit the virus replication [103].

Recently, Qu et al. [104] presented a circRNA vaccine against SARS-CoV-2 that elicited potent neutralising antibodies and T-cell responses. By expressing the trimeric receptor binding domain of the spike protein, it provides robust protection in both mice and rhesus macaques [104]. Notably, the circRNA vaccine against SARS-CoV-2 enabled higher and longer-lasting antigen production than the mRNA vaccine and elicited a higher percentage of neutralising antibodies and distinct Th1-type immune responses [104].

## 7. Conclusions

In the past, circRNAs were considered a “waste” of transcription, a by-product of abnormal splicing of RNA without a precise function. The discovery of circRNAs has revolutionised and is revolutionising current knowledge of the mechanisms underlying major biological processes. Today, increasing evidence shows that circRNAs are critical in multiple regulatory processes, ubiquitous, tissue- and cell-specific and even conserved in their sequences across the species.

In viral infections, circRNAs are strictly correlated with host biological process and cycle of the virus, thus behaving like biomarkers of infectious disease (undergoing down regulation or upregulation), by influencing the severity and extent of pathogenicity of the infectious agent, and through the synthesis of ncRNA.

Thus, knowing and recognising cell-encoded and virus-encoded circRNAs allows for the distinction between viral (i.e., circE7 encoded by HPV-16 [89]) and non-viral diseases (i.e., hsa_circ_0004277 identified as biomarker for acute myeloid leukaemia [105]), among the phases of diseases progression (i.e., hsa_circ_0015962 and hsa_circ_0006459 in Dengue virus infection [84]), and the molecular pathways in which circRNAs are involved (i.e., circBART2.2 encoded by EBV [91]).

The enormous potential of circRNAs and possible applications pave the way for the design of pioneering models aimed at knowing, studying, understanding and modulating the regulatory pathways they are involved. However, several gaps remain; it is still difficult to identify new circRNAs, to recognise their origin, to understand which mechanisms they are involved in, to define their interaction with complex molecular systems.

Furthermore, although a multitude of virus-encoded and cellular-encoded circRNAs are known to date, no specific standard nomenclature system has been established. This, together with the lack of standardised methods of study, lends to great confusion in sequences definition and in recognising/identification of circRNAs. A single database containing all information on the sequences, functions and mechanisms of action of circular RNAs should be established as soon as possible.

In conclusion, thanks to their stability, specificity, detectability in body fluids, circRNAs may become new targets for the development of innovative therapeutic strategies and innovative tools in diagnosis of several diseases, including viral infections, even though further studies are necessary to completely define their biological functions and application in diagnosis as biomarkers and in therapy as drugs.

## Figures and Tables

**Table 1:** Cellular-encoded circRNAs involved in viral infections and/or in virus-related diseases and their principal role.

circRNAs Involved	Virus or Virus-Related Diseases	Principal Role	References
hsa_circ_0003046	Herpes Simplex Virus 1	Not specified	[64]
hsa_circ_0003683			
hsa_circ_0007752			
hsa_circ_0007231			
hsa_circ_0006783			
hsa_circ_0100381	Hepatitis B Virus-related Hepatocellular Carcinoma	Not specified	[65]
hsa_circ_0103489			
hsa_circ_0101764			
hsa_circ_0104351			
hsa_circ_0102814			
hsa_circ_0001225			
hsa_circ_0102109			
hsa_circ_0101092			
hsa_circ_0102904			
hsa_circ_0100327			
hsa_circ_0000976		Not specified	[66]
hsa_circ_0007750			
hsa_circ_0139897			
hsa_circ_0006942		Induces Virus Replication and Expression by Regulating miR-138-5p/TNFAIP3 Axis	[67]
circRNA_100338		Regulatory Role of the circRNA-100338/miR-141-3p Pathway	[68]
hsa_circ_0067934		Promotes Tumour Growth and Metastasis by Regulation of miR-1324/FZD5/Wnt/β-catenin Axis	[69]
hsa_circ_0004812		Sponging miR-1287-5p	[70]
hsa_circ_0004018		Prognostic Biomarkers	[71]
hsa_circ_0003570			
hsa_circ_0005389	Chronic Hepatitis B	Not specified	[72]
hsa_circ_0000038			
hsa_circ_0000650			
circRNA_10156	Hepatitis B Virus-related Liver Cancer	Pro-tumorigenic Function	[73]
hsa_circRNA_001387	Epstein-Barr Virus-related Nasopharyngeal Carcinoma Cell	Biomarker	[74]
hsa_circ_0051620	Human Papillomavirus 16	Probably Implicated in the mTOR Signalling Pathway, Proline Metabolism and Glutathione Metabolism.	[75]
hsa_circ_0052602			
hsa_circ_0048867			
hsa_circ_0038475			
hsa_circ_0035918			
hsa_circ_0056353			
hsa_circ_0026527			
hsa_circ_0037213			
hsa_circ_0018289	Human Papillomavirus-related Carcinoma	Promotes the Tumorigenesis	[76]
hsa_circ_0024169		Diagnostic Biomarker of Angiosarcoma	[77]
hsa_circ_081069		Probably Involved in Tongue Squamous Cell Carcinoma Development	[78]
circ-GATAD2A	Influenza A Virus	Promotes Virus Replication by Inhibiting Autophagy	[79]
hsa_circ_0001400	Kaposi’s Sarcoma Herpesvirus	Antiviral Activity	[16]
hsa_circ_0001741			
hsa_circ_0008311			
hsa_circ_0005145			
hsa_circ_0001808		Promotes Oncogenesis	[80]
hsa_circ_0000711	Human Immunodeficiency Virus 1	Involved in the pathogenesis of Early Infection	[81]
hsa_circ_0006968			
hsa_circ_0003863			
hsa_circ_0049083			
hsa_SATB1_AS1_0002		Potentially Promote Viral Replication	[82]
hsa_ANKRD17_0008			
hsa_ATXN1_0001			
hsa_FAM13B_0019			
hsa_FBXW7_0005			
hsa_HIPK3_0001			
hsa_PHC3_0020			
hsa_ZNF609_0001			
hsa_circ_0001445		Promote Virus Transcription	[83]
hsa_circ_0000479	Hantaan Virus	Regulation of Viral Infection	[58]
hsa_circ_0015962	Dengue Virus	Biomarkers	[84]
hsa_circ_0006459			
hsa_circ_0001445	Human Cytomegalovirus	Potentially Promotes Viral Replication	[85]
hsa_circ_0001206			
hsa_circ_0067985	Middle East Respiratory Syndrome Coronavirus	Biomarkers	[86]
hsa_circ_0006275			

**Table 2:** Virus-encoded circRNAs involved in viral infections and their principal role.

Virus-Encoded circRNAs	Virus	Principal Role	References
circE7	Human Papillomavirus 16	Cells Replication	[89]
HBV_circ_1	Hepatitis B Virus	Progression of Hepatocellular Carcinoma	[90]
circBART2.2	Epstein–Barr Virus	Promote Immune Escape	[91]
circLMP-2_e5		Not specified	[92]
circRPMS1		Suppression Nasopharyngeal Carcinoma Cell Proliferation and Metastasis by Sponging miRNAs	[93]
circLMP2A		Induces Stemness in EBV-associated Gastric Cancer	[94]
circRPMS1_E4_E3a		Latency and Viral Replication	[95]
circRPMS1_E4_E2			
circEBNA_U			
circBART_1.1		Viral Oncogenesis	[96]
circBART_2.1			
circBART_1.2			
circvIRF4	Kaposi’s Sarcoma Herpesvirus	Viral Oncogenesis	[96]
circPAN/K7.3		Biomarker	[97]
